# Enhancing the Toughness of Free-Standing Polyimide Films for Advanced Electronics Applications: A Study on the Impact of Film-Forming Processes

**DOI:** 10.3390/polym15092073

**Published:** 2023-04-27

**Authors:** Ruoqing Zhao, Hao Wu, Xuan Dong, Manzhang Xu, Zhenhua Wang, Xuewen Wang

**Affiliations:** Frontiers Science Center for Flexible Electronics (FSCFE), Shaanxi Institute of Flexible Electronics (SIFE), Northwestern Polytechnical University, Xi’an 710072, China

**Keywords:** polyimide film, high toughness, performance regulation, fabrication process optimization

## Abstract

High-quality and free-standing polyimide (PI) film with desirable mechanical properties and uniformity is in high demand due to its widespread applications in highly precise flexible and chip-integrated sensors. In this study, a free-standing PI film with high toughness was successfully prepared using a diamine monomer with ether linkages. The prepared PI films exhibited significantly superior mechanical properties compared to PI films of the same molecular structure, which can be attributed to the systematic exploration of the film-forming process. The exploration of the film-forming process includes the curing procedures, film-forming substrates, and annealing treatments. Additionally, the thickness uniformity and surface homogeneity of free-standing films were crucial for toughness. Increasing the crystallinity of the PI films by eliminating residual stress also contributed to their high strength. The results demonstrate that by adjusting the above-mentioned factors, the prepared PI films possess excellent mechanical properties, with tensile strength and elongation at break of 194.71 MPa and 130.13%, respectively.

## 1. Introduction

Compared with traditional electronic devices, flexible micro/nano devices have garnered significant attention in recent years due to their flexibility and wearable characteristics [1]. These devices have found widespread use in healthcare, flexible storage, flexible displays, and other fields [2]. As the carrier of functional structure and the sensitive unit, the performance of flexible substrate directly determines the further thinness of flexible micro/nano devices [3]. Polyimide (PI) is one of the most commonly used flexible substrates due to its remarkable mechanical properties, excellent thermal stability, and low coefficient of thermal expansion [4,5]. The easily regulatable molecular structure of PI molecules has led to the development of various functional PIs, such as transparent PI, photosensitive PI, and recyclable PI [6,7,8]. Additionally, the mechanical properties of PI films have been further improved to meet more complex application environments, such as aerospace and military fields [9,10].

Nowadays, to meet the demand for PI films with excellent mechanical properties, different endeavors have been proposed to reinforce the PI films, including incorporating the inorganic fillers [11,12] and designing the chemical structure of monomers [13,14]. Generally, the incorporation of inorganic substances increases the tensile strength and modulus while reducing the elongation at break [15,16]. Besides, much more attention has been given to modifying the chemical structure of the polymer backbone. Yang et al. [17] designed a novel hyperbranched PI (HBPI) that increased the free volume of the system, resulting in a significant improvement in the toughness of the modified resin. However, the design and synthesis of monomer structures are often complex and time-consuming, which greatly limits the efficiency of PI film preparation. Recently, there has been a growing interest in studying the effect of the imidization process on enhancing the performance of PI materials, as the cost of novel diamines and dianhydride monomers is high and limits their commercial feasibility [18,19,20]. Ji et al. [21] traced and studied the structure changes from poly(amic acid) (PAA) fibers to polyimide fibers during ramp-heating and isothermal treatments. Chung et al. [15] improved the tensile strength by increasing the flexibility of PI films via microwave (MW) irradiation of the PAA. The improvement in mechanical properties can be attributed to the increase in molecular weight of the PI by MW irradiation. Nevertheless, there is currently a lack of systematic conditions exploration of the film formation process. For most PI films, achieving high mechanical strength and toughness simultaneously remains a challenge, as these properties tend to be mutually exclusive. Thus, the fabrication of PI films with outstanding comprehensive properties and a balance between toughness and strength is still a significant challenge.

In this study, the effect of film-forming conditions on the mechanical properties of thin films was systematically investigated, and a highly tough free-standing PI film was simply prepared from diamine monomers with ether linkages. The PAA solution was prepared by a two-step polycondensation method under the protection of a nitrogen atmosphere. The flexible and robust PI film was successfully prepared by the spin coating method. By systematically exploring the film-forming conditions, such as the rotation speed, thermal treatment process, and annealing treatment, high-toughness free-standing PI films were easily prepared using diamine monomers with ether linkages. These films exhibit excellent thickness uniformity and surface homogeneity, as well as outstanding mechanical properties, including both strength and toughness. The tensile strain behavior of PI films was analyzed at room temperature using a tensile test machine. The results revealed that the films exhibited outstanding mechanical properties, with a tensile strength of 194.71 MPa and elongation of 130.13%.

## 2. Materials and Methods

### 2.1. Materials

Pyromellitic dianhydride (PMDA) (98%) was provided by Tokyo Chemical Industry (TCI) (Shanghai, China). 1,4-Bis(4-aminophenoxy) benzene (TPE-Q) (98%) was purchased from Shanghai Aladdin Bio-Chem Technology Co., Ltd., Shanghai, China. N,N-Dimethylformamide (DMF) (99.8%, Extra Dry) was supplied by Energy Chemical (Shanghai, China). All chemicals are not treated before being directly used.

### 2.2. Characterization

IR spectra were taken on a Fourier transform infrared (FT-IR) spectrophotometer (Bruker Tensor II, Karlsruhe, Germany). Viscosity was measured with a Brookfield DV2THB viscometer in DMF at 30 °C. The measurements were conducted as promptly as possible to avoid the depolymerization of PAAs. Surface roughness was measured by Dimension Icon atomic force microscopy (AFM) of Bruker (Dimension Icon, Karlsruhe, Germany). The glass transition temperature of the PI film was measured by NETZSCH (DMA 242E, Selb, Germany) with a heating rate of 10 °C·min^−1^ from 30 °C to 480 °C under nitrogen. Mikrometry DHG-050 was used to measure the thickness of the films. A UV-vis spectrophotometer (Hitachi U-3900H, Tokyo, Japan) was used to measure the optical transmission. The scan range was 300–800 nm, and the scan rate of transmission measurement was 600 nm·min^−1^. The tensile strength and elongation at break of PI specimens with dimensions of 30 mm in length and 2 mm in width were measured using a CMT4103 universal testing instrument at room temperature and a testing speed of 10 mm min^−1^. The specimens were carefully cut from high-quality samples measuring 5 cm × 5 cm, which were free of any defects such as fine bubbles. X-ray diffraction (XRD) was executed in the range of 5–80° at a scanning speed of 8 (°)·min^−1^ on the Bruker (D8 Advance, Karlsruhe, Germany) with Cu Kα radiation (λ = 0.154 nm) at 40 kV.

### 2.3. Synthesis of PAA Precursor

**Scheme 1:**
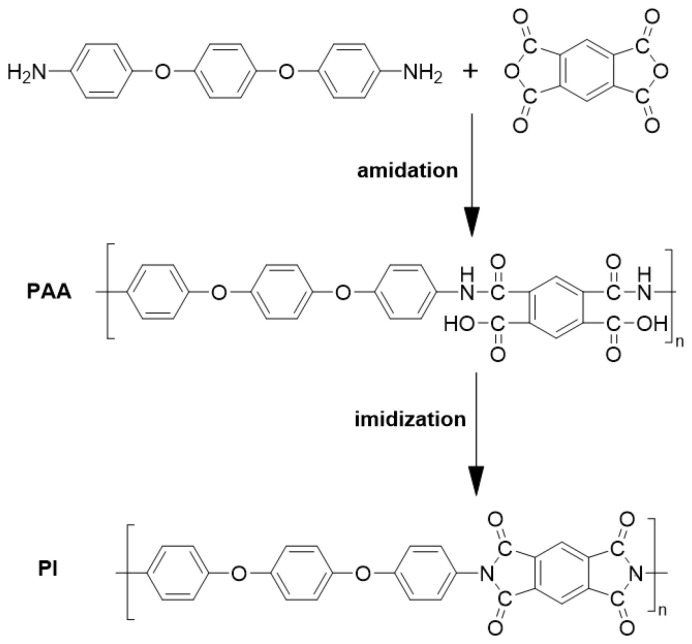
General synthesis route of polyimide.

**Figure 1:**
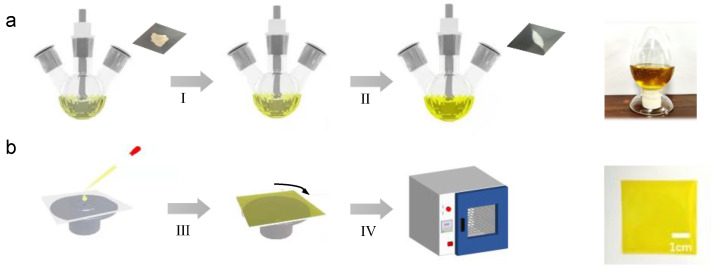
(**a**) Schematic illustration of PAA synthesis, (**b**) fabrication process of PI films.

Given that the focus of this article is on the influence of the preparation process on the mechanical properties of the films, we chose PMDA as the dianhydride monomer, which is the most widely studied. Additionally, to achieve the high toughness of the resulting PI films, we selected TPE-Q, a diamine monomer rich in ether bonds. There have been numerous reports in the literature on the synthesis of PMDA-TPE-Q [22,23,24,25,26]. In our study, we adopted a simple two-step method to prepare the PI films (Scheme 1). First, the diamine monomer with two ether bonds was reacted with dianhydride to obtain the corresponding PAA precursor and subsequently heat-treated to obtain the PI films by thermal imidization. Figure 1a shows the schematic illustration for the synthesis of PAA. 5.84 g of TPE-Q (0.02 mol, 1 equiv.) and 30 mL of DMF were added to the flask. The mixture was continuously stirred under an N_2_ atmosphere. Until the diamines were completely dissolved, 4.36 g of PMDA (0.02 mol, 1 equiv.) and 30 mL of DMF were weighed and added into the obtained pale yellow diamine solution by three times. Considering the possible hydrolysis of dianhydride, the addition of PMDA should be fast to avoid the introduction of moisture. The solution became viscous immediately after the PMDA was completely added. The reaction last for 2 h under the protection of N_2_ at room temperature to ensure full polymerization. Then, the viscous and homogeneous PAA solution was obtained.

### 2.4. Preparation of PI Films

As shown in Figure 1b, the PI films were prepared from PAA by spin coating. The spin coating speed was set to 1000 to 9000 rpm. Glass and Si/SiO_2_ were selected as substrates, in which glass substrates needed ultrasonic cleaning before use. After coating on a clean substrate, the PAA film was treated through a series of gradient curing in an oven at 70 °C for 1 h, 150 °C for 2 h, 200 °C for 1 h, and 250 °C for 1 h. After cooling to ambient temperature, the PI films were immersed in deionized water for 0.5 h, then peeled off from the substrates. They were washed with ethanol, and dried at room temperature for further use.

## 3. Results and Discussion

### 3.1. Controllable Fabrication of PI Films

#### 3.1.1. Chemical Structure and Stability of PAA

**Figure 2:**
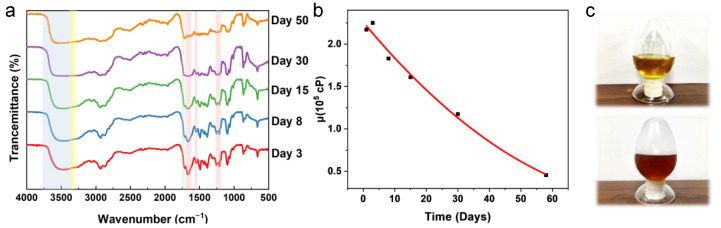
Changes of PAA solution with increasing storage time. (**a**) FT-IR spectra, (**b**) viscosity, (**c**) images of virgin and degraded PAA solution.

The molecular structures of PAA were confirmed by FT-IR. As shown in Figure 2a, a weak and broad peak at 3500 cm^−1^ was assigned to the hydrogen-bonded O–H. The peak at 3300 cm^−1^ was due to the N–H stretching vibration. Three characteristic absorption bands of PI at 1665 cm^−1^, 1548 cm^−1^, and 1255 cm^−1^ were found, which were attributed to the C=O asymmetrical stretching of imide groups, N–H vibration, and C–N stretching of the imide ring respectively. The results indicated that pure PAA was synthesized.

PAA serves as a crucial precursor in the production of PI films, and the quality of the PAA solution has a direct impact on the final performance of the PI films [27]. Unfortunately, PAA solutions are prone to instability upon storage and may undergo undesirable changes in viscosity [28]. The degradation of PAA can be attributed to two factors: firstly, the hydrolytic degradation of PAA due to the presence of trace water in the solvent, and secondly, the reversible formation of polymeric acid that causes chain length equilibrium in PAA [29,30]. For investigation of the stability of prepared PAA solutions, viscosity, and structure tests were carried out with a PAA solution stored with increasing time. It can be seen from the FT-IR spectra (Figure 2a) that the characteristic absorption band of the amide group gradually weakened, while the C=O absorption peak at 1714 cm^−1^ enhances during 30 days of storage. However, when the storage time exceeded 50 days, the characteristic peaks of the amide group changed significantly. It can be concluded that the PAA solution was still available within 30 days of storage, but may deteriorate after 50 days. Figure 2b shows the changes in the viscosity of the PAA solution with increasing storage time. The viscosity of the PAA solution gradually decreased with storage, which also predicted that the polyimide solution was gradually deteriorating. Based on the experimental results, it is important to store newly prepared PAA solutions at a low temperature (0–10 °C) and in a sealed container, as the effective storage period of PAA solutions is about 30 days. As shown in Figure 2c, the virgin PAA solution is presented as a clear yellowish liquid. With increased storage time, the water vapor in the air will inevitably penetrate the storage container, resulting in the hydrolysis and fracture of the amide bond of the PAA and the reduction of the degree of polymerization. Macroscopically, a darker discoloration, and thinning behavior was observed.

#### 3.1.2. Thickness and Uniformity Regulation of PI Films

**Figure 3:**
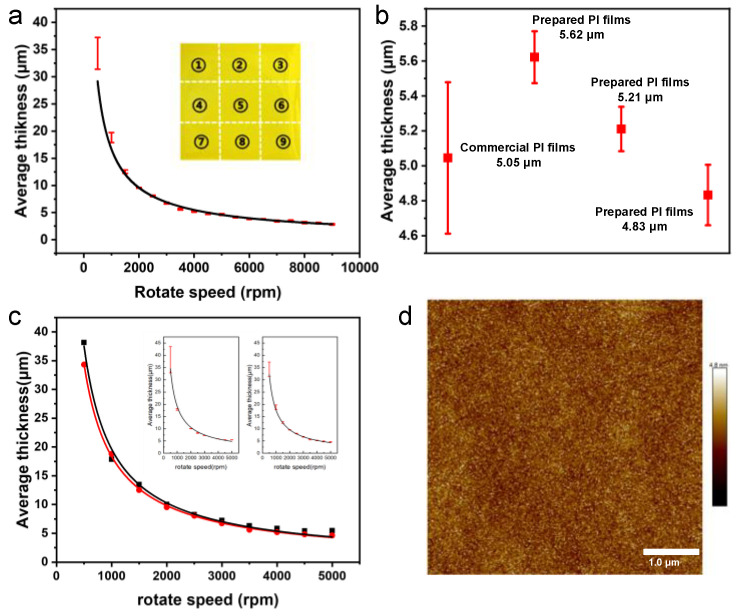
Results of thickness and uniformity of PI films. (**a**) PI films prepared by spin coating under different rotation speeds, (**b**) commercial films and similar thickness films prepared, (**c**) two different batches under the same process parameters, (**d**) AFM images of PI films.

The thickness of the synthesized PI film was tested by DHG-050 digital height gauge. Nine random sites on the film were selected for thickness testing, and the average value was taken as the thickness of the film. Film uniformity was evaluated as the variance of thickness values measured at nine randomly selected sites. As shown in Figure 3a, when PAA with a viscosity of 62,300 cPs was used for spin coating, the thickness of PI films gradually decreased with the increase in rotating speed. Uniform PI films with thicknesses ranging from 2.82 to 34.32 μm can be obtained by adjusting the rotating speed during spin coating. The film thickness changed obviously in the speed range of 500–3000 rpm. The prepared PI film exhibited excellent thickness uniformity, with an optimal uniformity of 0.77%. In comparison, commercially available films were also characterized for thickness and uniformity, as shown in Figure 3b. Compared to the commercially available films, the films prepared by this method with similar thickness showed significantly better uniformity. In addition, several fabrications were conducted with the same process parameters at 500–5000 rpm to verify the repeatability of the film-forming process. The representative results are shown in Figure 3c. Compared to the results of the control group, the repeated experiments showed only slight variations, indicating that the process has excellent controllability.

The surface morphology of the film obtained by AFM was shown in Figure 3d. When glass was used as the film substrate, the surface of the PI films was smooth, and the Ra (roughness average) of the surface was only 0.34 nm. The thickness uniformity and surface homogeneity of free-standing films were crucial for toughness.

#### 3.1.3. Residual Stress Elimination of PI Films

As a common phenomenon in the film fabricating process, residual stress will lead to the curling and cracking of films, and even result in the falling off of the films from the substrate, thus affecting the microstructure, mechanical, optical, and other physical properties of the film [31]. When PI films were used as flexible substrates, on the one hand, the curl caused by the residual stress will complicate the device preparation process; On the other hand, stress concentration caused by residual stress will lead to poor mechanical properties of devices. Annealing was an effective way to reduce the residual stress of thin films introduced in the preparation process. The PI molecular segment will relax when the imidization temperature reaches the glass transition temperature (*T*_g_) [32]. Irregular crystal domains melted during the annealing process, then larger crystal structures formed [33,34]. The process increased the crystallinity of polymers and reduced the free volume between polymer chains.

**Figure 4:**
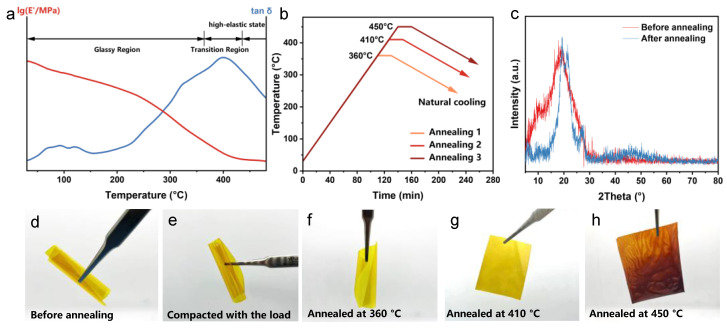
(**a**) DMA curves of PI film, (**b**) annealing procedure, (**c**) XRD patterns of PI film before and after annealing, (**d**) PI film before annealing, (**e**) PI film compacted with the load only, (**f**) PI film annealing at 360 °C, (**g**) PI film annealing at 410 °C, and (**h**) PI film annealing at 450 °C.

Figure 4a shows the dynamic mechanical analysis (DMA) curves of PI film. It can be observed that the storage modulus of the PI film drops sharply in the glass transition area until a stable platform (red curve). At the same time, the mechanical loss of the film reaches a maximum (blue curve) in the glass transition region. It can be determined that the *T*_g_ of the prepared film is about 400 °C. As shown in Figure 4b, the newly prepared PI film was cut into four splines and treated by different designed annealing processes. The crimped film was compaction with the load and placed in a muffle furnace, heated to 360 °C, 410 °C and 450 °C with the heating rate of 3 °C min^−1^, kept for 20 min, and then naturally cooled. Figure 4d shows the image of the PI spline before treatment, which was severely crimped. As shown in Figure 4f, the crimp magnitude of PI film was slightly improved when the annealing temperature (T_1_ = 360 °C) is lower than *T*_g_. When the annealing temperature (T_3_ = 450 °C) is excessively high, although the film became flat, it deteriorated (Figure 4h). Only when the annealing temperature (T_2_ = 410 °C) is about equal to *T*_g_, the film no longer curled (Figure 4g), which means that the residual stress was eliminated. In contrast, the film was still severely crimped if compaction with the load without annealing (Figure 4e). In conclusion, annealing the prepared crimped PI film with *T*_g_ as the annealing temperature is an effective way to reduce its residual stress and increase its flatness. The reduction of residual stress can increase the crystallinity of the PI film, thereby ensuring its high strength. As shown in c, significant XRD differences appeared in PI film through annealing. The annealed PI film presented sharper diffraction peaks compared with virgin PI film, suggesting its higher crystallinity.

### 3.2. Performance Regulation of PI Films

#### 3.2.1. Mechanical Properties

**Figure 5:**
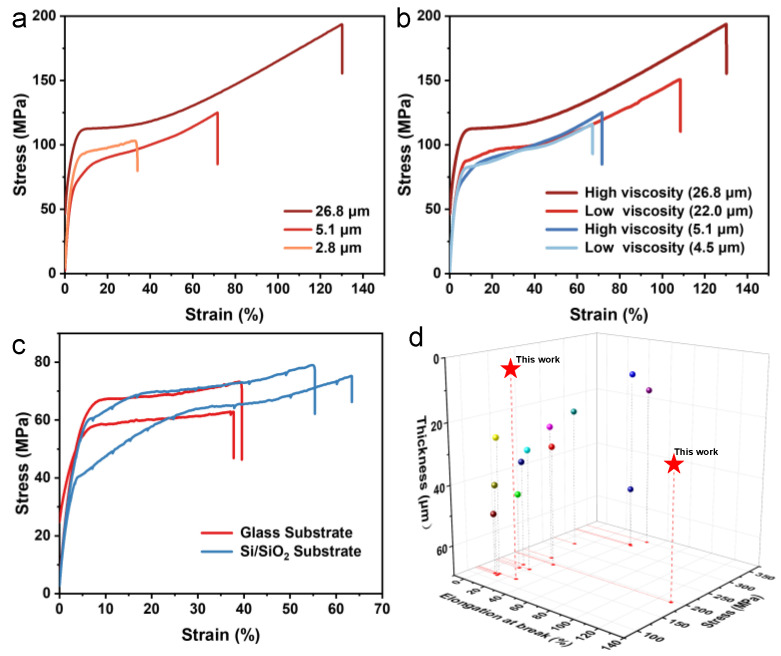
Stress-strain curves of PI films with different factors. (**a**) thickness, (**b**) viscosity, (**c**) substrate, (**d**) Comparison of the fracture elongation, tensile strength, and thickness of the prepared PI films with those reported in other studies [14,16,35,36,37,38,39,40,41,42,43,44].

The mechanical properties of the prepared PI films were characterized by a tensile testing machine. Figure 5a–c shows the tensile stress-strain curves of PI films under the influence of different factors. It can be observed that the prepared PI films exhibit excellent toughness. The excellent toughness of the prepared PI films can be partially attributed to the presence of a relatively large number of ether bonds in the molecular chain, which increases the number of allowed conformations and the flexibility of individual chains. As shown in Figure 5a, the tensile strength and elongation at break of the PI film increase with increasing film thickness. When the film thickness was 26.8 μm, the tensile strength and elongation at break reached 194.71 MPa and 130.13%, respectively. Additionally, the free-standing PI film that we prepared, with a thickness of only 2.8 μm, also exhibited excellent mechanical properties, with a tensile strength of 120.96 MPa and an elongation at break of 28.66%. The viscosity of the PAA precursor affected the thickness of the PI film, and therefore we hypothesize that it also has some influence on the mechanical properties of the film. As shown in Figure 5b, the mechanical properties of films prepared by spin-coating solutions with high viscosity (62,300 cP) and low viscosity (26,000 cP) were compared. It can be concluded that the difference in solution viscosity within a certain range has a negligible influence on the tensile strength and elongation at break of the film, indicating the potential to achieve thinner and lighter PI films with excellent mechanical properties. The mechanical properties of the films were greatly influenced by the substrate used for film formation. As depicted in Figure 5c, the films deposited on Si/SiO_2_ substrates exhibited superior toughness compared to those on glass substrates, possibly due to the lower surface roughness of Si/SiO_2_. Figure 5d shows the mechanical properties of PI films prepared in different studies [14,16,35,36,37,38,39,40,41,42,43,44]. Table 1 shows the structure, thickness, and corresponding mechanical properties of the PI films. It can be seen that the elongation at break of the PI film we prepared (26.8 μm) is much higher than other films, and the tensile strength is also comparable. Meanwhile, the free-standing PI film we prepared with a thickness of only 2.8 μm exhibited equally impressive in both strength and toughness. In addition, a comparison was made with other PI films of the same molecular structure in other studies [24,45,46], and the mechanical properties of the PI film we prepared exhibited significant superiority. Meanwhile, PI films with only the same diamine structure were also investigated [47,48,49], and the results also showed that the mechanical properties of the PI films we prepared were much better than others.

**Figure 6:**
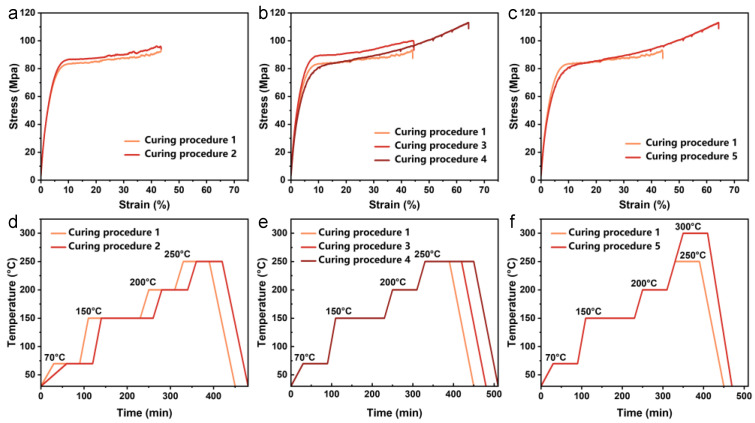
The effects of curing procedure on the mechanical properties of PI films. (**a**) Curing procedures with different solvent volatilization rates, (**b**) different imidization holding times, and (**c**) different imidization temperatures. (**d**) The strain–stress curves of PI films treated with different solvent volatilization rates, (**e**) different imidization holding times, and (**f**) different imidization temperatures.

To investigate the effects of solvent volatilization rate, imidization temperature, and imidization holding time on the mechanical properties of PI films during the heating process, we studied the tensile stress-strain curves of PI films treated by different temperature-programmed processes. As shown in Figure 6a, different heating methods were designed to construct different solvent evaporation processes to explore the influence of solvent evaporation rate on the mechanical property of PI film. The result shows that the aggregation structure of the PI film depends on the heating rate. Different heating rates will affect the solvent residue in the wet film and the degree of imidization of PAA. An extremely fast heating rate led to a fast solvent volatilization rate, which may cause bubbles and voids on the surface of the film, reducing its tensile strength (Figure 6d). In addition to the influence of solvent volatilization rate, the thermal imidization process was also essential to the mechanical properties of the films. The curing of PAA to produce the corresponding PI involves several thermal processes. As the temperature increases, both the solvent residue ratio and the degree of imidization are affected by the curing procedures. Therefore, different thermal imidization processes were designed to explore the influence of imidization temperature and holding time on the obtained PI films. Figure 6e shows the curing procedures (curing procedures 1, 3, and 4) for different imidization times at a certain temperature. It can be observed that the longer the thermal imidization time of the PI films, the higher the degree of imidization, and the easier it is for the molecular chains to form a compact and orderly stacking, leading to the formation of PI films with higher tensile strength and elongation at break (Figure 6b). Figure 6f shows two curing procedures with different imidization temperatures. It can be seen that when the imidization temperature was increased from 250 °C to 300 °C, the retained solvent was reduced, and simultaneously, the molecular chain activity was strong at high temperatures, leading to a high degree of imidization and the formation of PI films with stronger intermolecular interaction and higher tensile strength and elongation at break (Figure 6c).

#### 3.2.2. Optical Transparency

**Figure 7:**
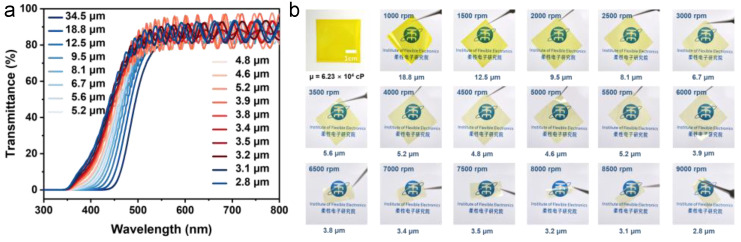
(**a**) The UV-vis spectra and (**b**) images of PI films with different thicknesses.

High optical transmittance is a crucial requirement for substrates and display devices [50]. The optical properties of the prepared PI films were analyzed by UV–vis spectrometry over a range of 300–800 nm (Figure 7a). The transparency of the films increased as the film thickness decreased. In addition, the films with different thicknesses have high transmittance in the visible light region, indicating the high transparency of the films. Figure 7b showed the images of PI films with different thicknesses, confirming the transparency changes with film thickness.

## 4. Conclusions

In summary, free-standing PI films with high toughness were prepared by a typical two-step polycondensation method. The prepared PI films exhibited great combined mechanical, thickness uniformity, and surface homogeneity, including tensile strength of up to 194.71 MPa, of elongation up to 130.13%, and *T*_g_ of 400 °C. The excellent mechanical properties of the films, including strength and toughness, are mainly attributed to two factors. The ether bonds in the diamine monomer endow the derived polyimide films with excellent toughness. On the other hand, systematic exploration of the film-forming process provides a method for achieving the high toughness of the films.

## Figures and Tables

**Table 1:** Composition and properties of PI films.

Dianhydride	Diamine	Filler	Thickness (μm)	Tensile Strength (MPa)	Elongation (%)	References
PMDA	ODA ^1^	OMMT ^13^	30	192	28	[35]
IMDA ^2^	ODA	/	45	153	14	[36]
BPDA ^3^	BOA ^4^	/	10	323.4	45.3	[37]
BTDA ^5^	ODA/DAPBI ^6^	/	30	157.8	21.5	[38]
BPDA	PABZ ^7^	/	25	210	17.9	[39]
ODPA ^8^	ODA	BP ^14^	25	119.6	11.5	[16]
Not provide	Not provide	Gh-BN ^15^	40	114	12	[40]
PMDA	ODA	GNR ^16^	35	165	12	[41]
BPDA	BIA ^9^/BPA ^10^	DPhP ^17^	16	350	50	[42]
BTDA	ODA	TapCzN ^18^	50	117	8.5	[43]
BPDA	PABZ	Cu^2+^	23	275	11	[44]
6FDA ^11^/BPDA	TFDB ^12^	/	50	326.7	43.2	[14]
PMDA	TPE-Q	/	26.8	194.71	130.13	This work
PMDA	TPE-Q	/	2.8	120.96	26.88	This work

^1^ 4,4′-Diaminodiphenyl ether. ^2^ 2,5-Bis(3,4-dicarboxyphenoxy)-1,4:3,6-dianhydromannitol dianhydride. ^3^ 3,3′,4,4′-Biphenyltetracarboxylic dianhydride. ^4^ p-Phenylenediamine. ^5^ 3,3′,4,4′-Benzophenonetetracarboxylic dianhydride. ^6^ 5,4′-Diamino-2-phenyl benzimidazole. ^7^ 5(6)-Amino-2-(4-aminobenzene)benzimidazole. ^8^ 4,4′-Oxydiphthalic anhydride. ^9^ 2-(4-Aminophenyl)-5-aminobenzimidazole. ^10^ 4,4′-diaminobiphenyl. ^11^ 4,4′-(Hexafluoroisopropylidene) diphthalic anhydride. ^12^ 2,2′-Bis(trifluoro-methyl)benzidine. ^13^ Organically modified-montmorillonite. ^14^ Black phosphorus nanoflakes. ^15^ Modified hexagonal boron nitride. ^16^ Graphene nanoribbon. ^17^ Diphenyl phosphate. ^18^ Tetraamino phthalocyanine zinc.

## Data Availability

Not applicable.
